# Age-Related Change in the Extent Physical Activity Attenuates Negative Affect Reactivity to Stressors in Daily Life

**DOI:** 10.1093/geroni/igab046.166

**Published:** 2021-12-17

**Authors:** Lizbeth Benson, Nilam Ram, David Conroy, Zita Oravecz, Timothy Brick, David Almeida

**Affiliations:** 1 The University of Oklahoma Health Sciences Center, United States; 2 Stanford University, Stanford, California, United States; 3 Pennsylvania State University, University Park, Pennsylvania, United States

## Abstract

Theories suggest that with increasing age, adults more effectively regulate their emotions and seek to limit high physiological arousal. Prior research indicates physical activity attenuates negative affect reactivity to stress, but also increases physiological arousal. The present study extends prior work by examining age-related differences and changes over time in the extent of attenuation. Participants (n=3,484; MedianAge=53.42 years, SD=13.3; 56% female), from the National Study of Daily Experiences completed 8 end-of-day assessments of their negative emotions, stress, and physical activity across 3 measurement bursts spaced approximately 10 years apart. Results from three-level multilevel models suggest that when full random effects are specified, physical activity does not attenuate negative affect reactivity to stress. Additionally, extent of attenuation did not differ with age or change over time. Discussion pertains to how these findings advance theoretical understanding of socioemotional development and to methodological nuances of random effects and non-normally distributed data.

